# Executive Functions Predict the Success of Top-Soccer Players

**DOI:** 10.1371/journal.pone.0034731

**Published:** 2012-04-04

**Authors:** Torbjörn Vestberg, Roland Gustafson, Liselotte Maurex, Martin Ingvar, Predrag Petrovic

**Affiliations:** 1 Department of Clinical Neuroscience, Karolinska Institutet Stockholm, Stockholm, Sweden; 2 School of Law, Psychology and Social Work, Örebro University, Örebro, Sweden; University of Granada, Spain

## Abstract

While the importance of physical abilities and motor coordination is non-contested in sport, more focus has recently been turned toward cognitive processes important for different sports. However, this line of studies has often investigated sport-specific cognitive traits, while few studies have focused on general cognitive traits. We explored if measures of general executive functions can predict the success of a soccer player. The present study used standardized neuropsychological assessment tools assessing players' general executive functions including on-line multi-processing such as creativity, response inhibition, and cognitive flexibility. In a first cross-sectional part of the study we compared the results between High Division players (HD), Lower Division players (LD) and a standardized norm group. The result shows that both HD and LD players had significantly better measures of executive functions in comparison to the norm group for both men and women. Moreover, the HD players outperformed the LD players in these tests. In the second prospective part of the study, a partial correlation test showed a significant correlation between the result from the executive test and the numbers of goals and assists the players had scored two seasons later. The results from this study strongly suggest that results in cognitive function tests predict the success of ball sport players.

## Introduction

Sport and Psychology have since the early 1920s been connected in a joint research area [1]. The focus for Sport Psychology has mainly been to understand and develop the performances among athletes in areas like motivation, group dynamics and mental training [2]. Another line of studies has focused on *talent identification*, in order to predict the success of an athlete [3]. These studies have investigated how personality *traits* or *states* correlate to successful sport behavior but no clear or consistent relationship has been demonstrated [4]. Multivariate analyses on ball-sport players have also been performed with variables like somatotype, body composition, body size, speed, endurance, performance measures, technical skill, anticipation, anxiety and task and ego orientation [5]. However, as for personality traits, no clear correlations between these variables and sport success have been established.

Apart from physical skills and basic coordination, success in ball-sports also depends on how information is processed given the complex and quickly changing contexts. In the last two decades a wide range of perceptual-cognitive skills have been studied in sport. This research has mainly focused on areas like visual anticipation, pattern recognition, knowledge of situational probabilities, and strategic decision-making [6]. This line of research has mostly studied sport-specific tasks [7], [8] comparing experts with novices and has contributed to the understanding of sport specific demands. A weakness in these studies is that expert and novice players cannot be compared with a neutral standard, with players in different sports or players in the same sport where other tests have been used. Thus, different studies are hard to compare and it is hard to understand how skills transfer across different types of sports [9]. This is of a general interest since it has been shown that experts in different sports are able to transfer cognitive skills between sports that make them more successful in the new sport then novices [10], [11].

Many required skills in team sports may be translated to general cognitive domains where test results from successful players can be compared to a population norm. A good team player could be characterized by excellent spatial attention, divided attention, working memory and mentalizing capacity. He or she must be able to quickly adapt, change strategy and inhibit responses. Many of these abilities are referred to as “game intelligence" in sports [12]. In neuropsychology these are collectively referred to as executive functions [13]. These dynamic cognitive top-down processes correlate with each other but less with general IQ [14]. Surprisingly, the impact of general executive functions on the capacity of a player is largely unknown [15]. One of few published studies on general cognitive skills in sports shows that there is a positive relationship between successful sport performances in young soccer players and their cognitive creativity in general [16].

Another problem in most research focusing on sport and cognition is the cross-sectional approach and the involvement of a sport specific situation that needs sport specific assessment tools made particularly for that study. The cross-sectional approach makes it difficult to establish any causal relation and the sport specific tools make it difficult and expensive to repeat the research in new studies.

In the present study we have explored the importance of general executive functions when it comes to predicting a successful outcome of a soccer player using both a cross-sectional and a prospective component. The study was divided in two parts and our approach was to use well-known neuropsychological assessment tools and assess the soccer players' executive functions such as the chain of creativity, working memory, multi-tasking and inhibition. We chose the Design Fluency test from the D-KEFS executive test battery as our primary test since it does not have a verbal component but include a creativity/planning aspect that we believe is important in team sports [6]. In order to strengthen our findings we also used two other executive tests from the same test battery (*Colour-word interference test* and *Trail making test*). In the first part we compared the results between High Division players (HD), Lower Division players (LD) and a standardized norm group. In the second part, two seasons later, we compared the test results with a well-known measure of success in soccer, namely the number of goal and assist - an objective measurement that characterizes the capacity of a team and a player without subjective valuations.

## Methods

### Ethics Statement

The study protocol, including the ethical aspects of the study, was approved by the Student Review Board at the Psychological Department of Örebro University. The study was performed in full compliance with the Declaration of Helsinki. All subjects were given verbal and written information on the study and gave their verbal and written informed consent to participate.

### Participants

The participants in the first (cross-sectional) part of the study included 57 male (n = 31) and female (n = 26) players (Table 1). 14 male and 15 female participants from the Swedish highest national soccer leagues (*Allsvenskan*) were included in the highest division group, HD (*M*_age_ = 25.3; SD: 4.2). 17 male participants playing in the Swedish 3^rd^ national division (called *Division 1*) and 11 female participants from Swedish 2^nd^ national division were included in the lower division group, LD (*M*_age_ = 22.8; SD: 4.1). There was no significant difference in age or educational level between the different groups (see Table 1).

**Table 1:** Descriptive table of the four included groups: High Division Males (HD-M), High Division Females (HD-F), Low Division Males (LD-M), and Low Division Females (LD-F).

Group	N	Position - Forw/Mid/Def	Age - Mean age (SD)	Higher Education - Mean years (SD)
HD-M	14	6/5/3	25,00 (4,87)	0.75 (1.11)
HD-F	15	4/9/2	25,60 (3,60)	1.87 (1.42)
LD-M	17	4/6/7	23,24 (3,05)	0.59 (1.33)
LD-F	11	4/4/3	22,18 (5,53)	1.05 (1.43)

There was no significant difference in age between HD-M and LD-M (t(29) = 1.232; p = 0.223), or HD-F and LD-F (t(24) = 1.911; p = 0.068). Likewise, there was no significant difference in higher education between HD-M and LD-M (t(29) = 0.364; p = 0.72), between HD-F and LD-F (t(24) = 1.448; p = 0.16), or between HD and LD players if the players were not divided by gender (t(55) = 1.541; p = 0.129.

The test group in the second (prospective) part of the study included 25 of the male players (13 from HD and 12 from LD at the time of testing) that had played at least one game in 1^st^ or 2^nd^ division since no official points were registered for the female players or players in division 3.

The participants for the HD group came from six teams of the highest division in Sweden, *Allsvenskan*. These teams represent 20% (♂) to 25% (♀) of the teams in their divisions. In the end of the season their average position in the final table was place nine (♂) of fourteen and place six (♀) of twelve. The participants for the LD group came from five *of the lower division* teams. These teams represent 20% (♂) to 17% (♀) of the teams in their divisions. In the end of the season their average position in the final table was the place five (♂) of fourteen and place five (♀) of twelve. The coaches from these teams were responsible to select the participants based on how well the soccer capacity of the individual player represented the team average level. The coaches were asked to select two forwards, two midfielders and two defenders from their teams and also asked not to select players where the probability was large of soon transferring to a higher or lower league. In average the players were participating in 70% of the games during the last 2.5 years.

### Materials

The tests used were from the D-KEFS test battery of executive functions where the scoring is normalized for age. The primary test used was *Design Fluency* (*DF*), a standardized test which measures on-line multi-processing such as creativity, response inhibition, and cognitive flexibility [17], [18] and thus simulates the executive chain of decision making in a similar way as in a real sport situation. DF is a non-verbal psychomotor test in which the participant uses the hand and a pen to combine all dots in a square with one line. The task is to find as many different combinations as possible of binding together the dots under time pressure (60 sec) and the participant is not allowed to use a solution twice. The participant needs to remember previous responses in an online working memory and update new rules accordingly (i.e. not repeat previous combinations). He or she must use inhibition skills in order not to repeat previous responses. The participant also needs to constantly use a scanning skill to find new solutions to fulfill the task. All three subtests of DF were used. As additional tests *Colour-word interference test* (CWI), i.e. *Stroop test*, and *Trail making test* (TMT) were used in order to confirm the result from of the primary test. These tests also measure general executive functions, but from a more verbal aspect and without the creativity or problem solving abilities aspects important in *DF.* Therefore, they are not optimal in the present analysis but serve as a control to the main test.

### Procedure

All the participating teams were visited at their training facilities from 7 June to 30 October 2007. The selected players were tested in a 40 minutes standardized process and with the same test leader.

### Design

The players were tested on their executive functions (fall 2007). Prospective data of goal and assist was used (January 2008 to May 2010) to study whether *DF* measured in 2007 could predict the outcome of a soccer player's success.

### Statistic analysis

A 2-way full factorial ANOVA with division and gender as factors, and scores on the executive test as dependent variable were used. Importantly, DF was used as our main analysis, as it was the only one of the D-KEFS tests containing factors we believe are important in soccer (fast creativity or problem solving abilities) and at the same time did not contain verbal aspect that may be highly affected by education/schooling (such as CWI and TMT). Thus, CWI and TMT are specifically used to strengthen the DF analysis. In order to be sure that other variables such as age, position and education did not affect the result we also performed an additional ANCOVA-analysis with the factors: division, gender and position, and the covariates: educational level and age. Scores on the DF test were treated as dependent variable.

In the prospective part a partial correlation between *DF* and square root of the points (goals and assists) controlling for the order in the team i.e. forward, mid-player or defender (given the different probabilities of scoring points) defined as two dummy variables, the proportion played in 2^nd^ vs. 1^st^ division (given that it is easier to score points in the 2^nd^ division) and the age (given both general physical decline and possible cognitive decline specifically in soccer) were used.

## Results

### Cross-sectional tests

#### *DF*

Using the sum of scaled scores male and female soccer-players in both HD and LD performed highly above the standard population in average on *DF* (Male HD: +1.93 SD, Female HD: +1.76 SD, Male LD: +1.02 SD, Female LD: +1.12 SD). Thus, HD-players belong to the 5% best individuals in the population on this test.

**Figure 1:**
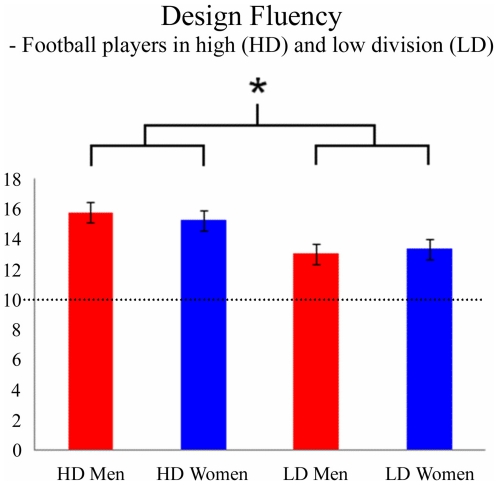
In Design Fluency (DF) the High Division (HD) players had significantly better scores than the Low Division players (LD). This difference was observed for both men and women. Note that both HD and LD players have superior scores compared with the standard population.

The ANOVA-model indicated a significant effect on *DF*-scores F(3, 53) = 4.99, *p* = 0.004. There was a significant effect of division (HD: mean-score: 15.52, SD: 2.42; LD: mean score: 13.18, SD: 2.14; F(1, 53) = 13.86; *p*<0.0005) but not of gender (F(1, 53) = 0.03; *p* = 0.86) or any interaction effect (F(1, 53) = 0.44; *p* = 0.51) (Figure 1). The effect was still present when also position, age and education-level were controlled for (ANCOVA-analysis F = 9.51; p = 0.004). No other effects were significant in the ANCOVA-analysis.

#### *CWI*

There was a trend for HD>LD in the *CWI* 1/2 (HD: mean-score: 11.62, SD: 1.57; LD: mean score: 10.86, SD: 1.94, F(1, 53) = 2.76, *p* = 0.10) and *CWI* 3 (HD: mean-score :12.48, SD: 1.79; LD: mean-score: 11.68, SD: 1.81; F(1, 53) = 2.57, *p* = 0.12) that are less demanding in terms of response suppression and response shifts. The more demanding test of executive functions, i.e. *CWI* 4, showed a significant difference between HD and LD (HD: mean-score: 12.17, SD: 1.98; LD: mean score: 10.79, SD: 2.79; F(1, 53) = 4,28, *p* = .044). Thus, only when there was a larger requirement of executive functions there was a better performance in the highest league vs. the lower league in this test.

#### *TMT*

In the part of the *TMT* which is testing sub-components important for executive functions including visual scanning, number sequencing, letter sequencing, and motor speed (i.e. *TM* 2/3) the HD group showed a significantly better performance compared with the LD group (HD: mean-score: 13.07, SD: 1.75; LD: mean score: 11.54, SD: 2.52; F(1, 53) = 6.73, *p* = 0.012). Importantly, the HD group had significantly higher points than the LD group on the primary executive component, i.e. *TMT* 4 (HD: mean-score: 11.69, SD: 1.47; LD: mean score: 10.68, SD: 1.66; F(1, 53) = 4.6; *p* = 0.037).

#### Correlation

*DF* correlated significantly with *CWI* 1/2 (*r* = 0.364; *p* = 0.005), *CWI* 3 (*r* = 0.414; p = 0.001), *CWI* 4 (*r* = 0.428; *p* = 0.001), *TM* 2/3 (*r* = 0.382; *p* = 0.003), *TM* 4 (*r* = 0.339; *p* = 0.011).

### Prospective test

In the prospective partial correlation test the *DF* result from 2007 was significantly correlated to the points (expressed as the square root of the points due to a skewed distribution) made January 2008 to May 2010, Correlation cf = 0.54; *p* = 0.006 (1-tailed).

## Discussion

This study shows that general executive functions are important in soccer and can even predict a future success in soccer players. In our cross-sectional test on executive functions (including our primary test, DF, and the two other control tests - CWI and TM) we found that the soccer players in the HD group had significant better results than soccer players in the LD group. Moreover, both groups performed much better on the executive tests than the general population. The findings were observed for both male and female players. The results are in line with previous studies on specific sport skills that indicate that elite athletes compared with sub-elite or novice has superior cognitive performance when it comes to sport specific situations [8]. Here we have been able to extend this finding to general executive functions but also to compare performance of both groups to a general population. In the prospective part of the study we showed that the DF predicted future success measured in goals and assists, suggesting a causal role for executive functions for sport success in soccer.

Executive functions are not a uniformly defined term but generally used as a term to describe the cognitive processes that regulate thought and action, especially in non-routine situations [19]. Examples of these processes are problem solving, planning, sequencing, selective and sustained attention, inhibition, utilization of feedback, multi-tasking, cognitive flexibility and ability to deal with novelty [20]. Different theoretical models are used to describe the executive functions. Relevant for this study are the supervisory attentional system (SAS) model [21] and the working memory model of Baddeley [22], since they both emphasize the global cognitive control operations of executive functions. The SAS model suggests a regulatory mechanism divided into two interactive components, the contention scheduling and the supervisory attentional control. Contention scheduling monitors the routine and over-learned behaviors while supervisory attentional control is responsible for monitoring new data and what is not yet routine. SAS has further been developed to the theory of multi-tasking performance in everyday life [23]. The executive functions have also been described as a fundamental part of working memory as the central executive component [24] interacting with the phonological loop and visual spatial sketchpad [22]. New information will be on-line analyzed and compared with earlier stored information to provide guidance to decision. The ability to use and update memory in order to predict future actions is a key aspect of the executive functions [25].

The development of the executive functions is considered to take place progressively throughout childhood and the adolescence from birth to 19 years of age [26], [27]. It is possible that good players actually develop better executive functions, although these functions have been regarded as relatively stable through life [28]. Executive functions are related to only some aspects of IQ [14], i.e. while the ability to update information in working memory is closely correlated with IQ, inhibition and quickly switching between different data show little or no relation to IQ.

The executive functions are thus important in order to capture and discriminate among information in decision-making, especially during time constraints. In ball-sports like soccer there are large amounts of information for the players to consider in every new moment. The successful player must constantly assess the situation, compare it to past experiences, create new possibilities, make quick decisions to action, but also quickly inhibit planned decisions. Thus, several core-features of executive functions such as planning, sustained and divided attention, suppression of previous responses, and working memory capacity are important for a team player in soccer. These executive functions are assessed in the tests used in the present study.

Executive functions - as a part of specific task related perceptual-cognitive functions - have previous been extensively studied in cognitive sports psychology for specific sports. For example it has been shown that expert soccer players can recall and recognize patterns of play more effectively than inexperienced soccer players [29] and that expert players in general have superior visual discrimination in a game-like situation [30], [31]. Studies on situational probabilities show that elite soccer players are better than their sub-elite counterparts in predicting and ranking the “best passing options" available [32]. Thus, they anticipate future events more efficiently, but also use this information to seek and pick up new information, and use different search strategies in different contexts of the play [29]. Similar studies in other ball sports [33] suggest that players evaluate the probability of each possible event that could occur and then use this information to maximize the efficiency of subsequent behavior. Research from basketball and field hockey [34], [35] also points out the importance of cognitive evaluation and that elite players have better ability than sub-elite players when it comes to recall of other players' position in a specific game situation. These studies are in line with the suggestion that expert players are superior in executive functions compared with novice players. However, they cannot isolate specific executive functions nor relate it to the general population. On a more theoretical level the suggestion that top-players are superior in general executive functions may change how the relation between cognition and sport success is conceptualized.

A possible shortcoming of the prospective part of this study is that we used goals and assists as our measures of performance quality and achievement. Thereby, this analysis may miss other factors that are not measured - for example how well did different individuals defend or organize the early game forward. We hope that we have been able to partially control for this as we controlled for position in our analyses. Nevertheless, goals and assists are easy to measure and undisputable. Thus, they are a good approximation of performance quality if other factors are well controlled for.

Investment in soccer players is a risky business where predictive tools are lacking. This study suggests that the precision in selecting the future stars should include not only judgement of physical capacity, ball control and how well the player performs at present. Our data suggest that measures of executive functions with validated neuropsychological tests may establish if a player has the capacity to reach top levels in soccer. Thus, the present study may change the way ball-sports are viewed and analysed and how new talents are recruited.
